# Simultaneous Estimation of Amlodipine Besylate and Indapamide in a Pharmaceutical Formulation by a High Performance Liquid Chromatographic (RP-HPLC) Method

**DOI:** 10.3797/scipharm.1203-07

**Published:** 2012-04-30

**Authors:** Deval B. Patel, Falgun A. Mehta, Kashyap K. Bhatt

**Affiliations:** Department of Pharmaceutical Chemistry and Analysis, Indukaka Ipcowala College of Pharmacy, New Vallabh Vidyanagar – 388121, Gujarat, India.

**Keywords:** RP-HPLC, Amlodipine, Indapamide, Tablet, Validation

## Abstract

An isocratic reversed-phase liquid chromatograpic assay method was developed for the quantitative determination of amlodipine besylate (AML) and indapamide (IND) in combined dosage form. A Brownlee C-18, 5 μm column with a mobile phase containing 0.02 M potassium dihydrogen phosphate–methanol (30+70, v/v) total pH-adjusted to 3 using *o*-phosphoric acid was used. The flow rate was 1.0 mL min^−1^ and effluents were monitored at 242 nm. The retention times of amlodipine besylate and indapamide were 5.9 min and 3.6 min, respectively. The proposed method was validated with respect to linearity, accuracy, precision, and robustness. The method was successfully applied to the estimation of amlodipine besylate and indapamide in combined tablet dosage forms.

## Introduction

Amlodipine besylate (AML) is chemically 3-ethyl 5-methyl 2-[(2-aminoethoxy)methyl]-4-(2-chlorophenyl)-6-methyl-1,4-dihydropyridine-3,5-dicarboxylate benzenesulfonate. It is a long-acting calcium channel blocker (dihydropyridine class) used as an anti-hypertensive and in the treatment of angina. It is official in IP, BP.

Indapamide (IND) is chemically 4-chloro-*N*-(2-methyl-2,3-dihydro-1*H*-indol-1-yl)-3-sulf-amoylbenzamide. It is a non-thiazide sulphonamide diuretic drug marketed by Servier, generally used in the treatment of hypertension, as well as decompensated cardiac failure.

At present, these drugs are available in combination therapy. The rationale behind use of this drug combination is that in treatment of hypertension in patients whose blood pressure is not adequately controlled by monotherapy, oral administration of amlodipine besylate and indapamide has been found to be more effective than use of either drug alone. Combination treatment with Amlodipine besylate and indapamide effectively reduces blood pressure in elderly patients with essential hypertension [1–5].

Literature survey revealed various spectrophotometric [6–9], HPTLC [10, 11] and HPLC [12–15] methods have been reported for estimation of AML individually or in combination with other drugs. Different spectrophotometric [16–18], HPTLC [19] and HPLC [20] methods have been reported for estimation of IND individually or in combination with other drugs. To the best of our knowledge, no analytical methods have been reported for analysis of AML and IND in pharmaceutical formulations. Hence the aim of the present study is to establish an accurate and sensitive RP-HPLC method and, after validation in accordance with International Conference on Harmonization (ICH) guidelines, to use the method for analysis of the drug content of both in tablet dosage form.[5]

## Results and discussion

### Optimization of mobile phase

**Fig. 2:**
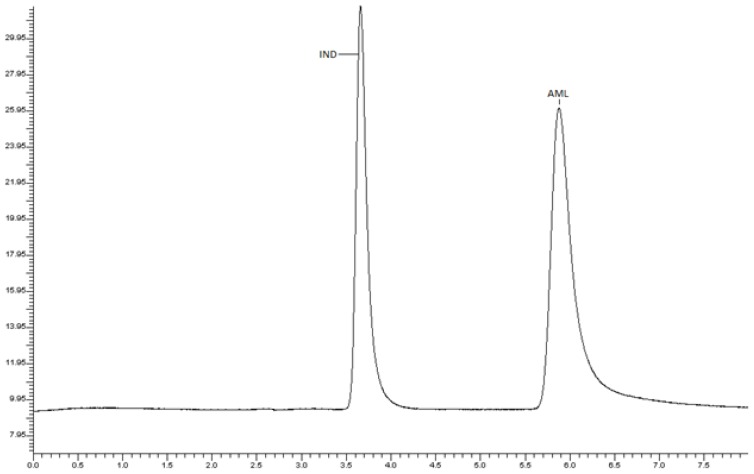
HPLC chromatogram of IND (RT 3.6 min) and AML (RT 5.9 min), using 0.02 M KH_2_PO_4_-methanol (30+70, v/v) total pH adjusted to 3 using O-phosphoric acid as mobile phase

Various mixtures containing methanol, water, ACN and aqueous buffer were tried as mobile phases in the initial stage of method development. Finally, the system containing mixture of 0.02 M KH2PO4-methanol (30+70, v/v) total pH adjusted to 3 using *o*-phosphoric acid was found to be satisfactory and gave two well-resolved peaks for AML and IND. The retention time for AML and IND were 3.6 min and 5.9 min, respectively (Fig. 2). The resolution between AML and IND was found to be 2.0, which indicates good separation of both the compounds. The mobile phase flow rate was maintained at 1 mL/min. Overlaid UV spectra of both the drugs showed that AML and IND absorbed appreciably at 242 nm, so detection was carried out at 242 nm.

### Validation of the Proposed Methods

The developed method was validated for various parameters including linearity and range, accuracy, precision, robustness, system suitability, specificity, LOQ and LOD.

#### Linearity and Range

The calibration curve for AML was found to be linear in the range of 0.25–35 μg mL^−1^ with a correlation coefficient of 0.9951. The calibration curve for IND was found to be linear in the range of 0.075–10.5 μg mL^−1^ with a correlation coefficient of 0.9945. The standard deviation value of slope of AML and IND were 101.25 and 739.78 which indicated a strong correlation between peak area and concentration. The regression analysis of calibration curves are reported in table 1.

#### Precision

Instrument precision was determined by performing injection repeatability test and the RSD values for AML and IND were found to be 0.18–0.63% and 0.61–0.79%, respectively. The intra-day and inter-day precision studies were carried out and the low RSD values indicate that the method is precise.

#### Accuracy

The accuracy of the method was determined by calculating recoveries of AML and IND by method of standard addition. The recoveries were found to be 99.46–101.60% and 97.33–100.88% for AML and IND, respectively (table 3). The high values indicate that the method is accurate.

#### Limit of detection and limit of quantification

The LOD and LOQ were measured by using an equation. The detection limits for AML and IND were 2.9 ng mL^−1^ and 0.99 ng mL^−1^, respectively, while quantitation limits were 8.8 ng mL^−1^ and 3 ng mL^−1^, respectively. The above data shows that a nano gram quantity of both drugs can be accurately and precisely determined.

### Stability of standard and sample solutions

Stability of standard and sample solution of AML and IND were evaluated at room temperature for 48 h. The relative standard deviation was found to be below 2.0%. It showed that both standard and sample solution were stable up to 48 h at room temperature.

### Specificity

The specificity study was carried out to check the interference from the excipients used in the formulations by preparing synthetic mixture containing both the drugs and excipients. The chromatogram showed peaks for both the drugs without any interfering peak and the recoveries of both the drugs were above 98%.

### Robustness

The method was found to be robust, as small but deliberate changes in the parameters of the method have no detrimental effect on the method performance as shown in Table 4. The low value of relative standard deviation indicated that the method was robust.

### Analysis of marketed formulations

A total of 20 tablets were accurately weighed and powdered in a mortar. An amount equivalent to one tablet (Containing 5 mg of AML and 1.5 mg of IND) was transferred to 25 mL volumetric flask, 10 mL of methanol was added and content of the flask were ultrasonicated for 10 min. The solution was filtered through whatman filter paper No. 41 and filter paper was washed with methanol twice and volume was then made up to the mark with methanol. The sample solution thus prepared was diluted with methanol to get the solution containing AML and IND in 5:1.5 μg/ml proportion, respectively. The solution was injected at above chromatographic conditions and peak areas were measured. The quantification was carried out by keeping these values to the straight line equation of calibration curve.

## Conclusion

This developed and validated method for simultaneous analysis of amlodipine besylate and indapamide in pharmaceutical preparations is very simple, rapid, accurate and precise. The method was successfully applied for determination of AML and IND in its pharmaceutical formulations. Moreover, it has advantages of short run time and the possibility of analysis of a large number of samples, both of which significantly reduce the analysis time per sample. Hence, this method can be conveniently used for routine quality control analysis of AML and IND in their pharmaceutical formulations.

## Experimental

The Liquid chromatographic system consisted of Perkin-Elmer HPLC model (VP series) containing LC-10AT (VP series) pump, variable wavelength programmable UV/VIS detector and Rheodyne injector with 20 μL fixed loop. The analytes were monitored at 242 nm. Chromatographic analysis was performed on Brownlee C-18 column having 250 × 4.6 mm i.d. and 5 μm particle size. All drugs and chemicals were weighed on Shimadzu electronic balance (AX 200, Shimadzu Corp., Japan).

### Chemicals and Reagents

Analytically pure samples of AML and IND were obtained as a gift samples from Alembic Pharmaceuticals Ltd (Baroda, India) HPLC grade methanol obtained from E. Merck Ltd., Mumbai, India while analytical reagent grade *o*-phosphoric acid and potassium dihydrogen phosphate were obtained from Astron Chemicals, India. Tablet formulation A (Amlodac-D, Zydus cardiva., India) and formulation B (NatrilAM, Serdia Pharmaceuticals Ltd., India) containing labeled amount of 5 mg of AML and 1.5 mg of IND, were procured from local market.

### Chromatographic conditions

A Brownlee C-18 (250×4.6 mm i.d) chromatographic column equilibrated with mobile phase 0.02M potassium dihydrogen phosphate–methanol (30+70, v/v) total pH adjusted to 3 with *o*-phosphoric acid (1M) was used. Mobile phase flow rate was maintained at 1 mL min^−1^ and effluents were monitored at 242 nm. The sample was injected using a 20 μL fixed loop, and the total run time was 10 min.

### Preparation of standard stock solutions

AML and IND were weighed (25 mg each) and transferred to two separate 25 ml volumetric flasks and dissolved in methanol. Volumes were made up to the mark with methanol to yield a solution containing 1000 μg mL^−1^ of AML and IND, respectively. Aliquot from the stock solution of AML was appropriately diluted with mobile phase to obtain working standard of 100 μg mL^−1^ of AML and same way for IND.

### Method Validation

The developed method was validated for various parameters like linearity and range, accuracy, precision, robustness, system suitability, specificity, LOQ and LOD.

#### Linearity and Range

Appropriate aliquots of AML and IND working standard solutions were taken in different 10 ml volumetric flasks and diluted up to the mark with mobile phase to obtain final concentrations of 0.25, 0.5, 2, 5, 10, 20 and 35 μg mL^−1^ of AML and 0.075, 0.15, 0.6, 1.5, 3, 6 and 10.5 μg mL^−1^ of IND, respectively. The solutions were injected using a 20 μL fixed loop system and chromatograms were recorded. Calibration curves were constructed by plotting average peak area versus concentrations and regression equations were computed for both drugs.

#### Precision

The intra-day and inter-day precision studies were carried out by estimating the corresponding responses 3 times on the same day and on 3 different days for three different concentrations of AML (2,5,10 μg mL^−1^) and IND (0.6,1.5,3 μg mL^−1^), and the results are reported in terms of relative standard deviation. The instrumental precision studies were carried out by estimating response of 3 different concentrations of AML (2,5,10 μg mL^−1^) and IND (0.6,1.5,3 μg mL^−1^), six times and results are reported in terms of relative standard deviation.

#### Accuracy

The accuracy of the method was determined by calculating recoveries of AML and IND by method of standard additions. Known amount of AML (0, 2.5, 5, 7.5 μg mL^−1^) and IND (0, 0.75, 1.5, 2.25 μg mL^−1^) were added to a pre quantified sample solution (containing AML and IND in 5:1.5 μg/ml proportion, respectively), and the amount of AML and IND were estimated by measuring the peak areas and by fitting these values to the straight-line equation of calibration curve.

#### Detection limit and Quantitation limit

The limit of detection (LOD) is defined as the lowest concentration of an analyte that can reliably be differentiated from background levels. Limit of quantification (LOQ) of an individual analytical procedure is the lowest amount of analyte that can be quantitatively determined with suitable precision and accuracy. LOD and LOQ were calculated using the following equation as per ICH guidelines.

LOD=3.3×σ/S; LOQ=10×σ/S;

Where σ is the standard deviation of y-intercepts of regression lines and S is the slope of the calibration curve.

#### Solution stability

Stability of sample and standard solution were stable up to 48 h at room temperature.

#### Specificity

Specificity is the ability of the method to measure the analyte response in the presence of its potential impurities and degradation products. Commonly used excipients (starch, microcrystalline cellulose and magnesium stearate) were spiked into a pre weighed quantity of drugs. The chromatogram was taken by appropriate dilutions and the quantities of drugs were determined.

#### Robustness

Robustness of the method was studied by deliberately changing the experimental conditions such as flow rate and percentage of organic phase.

## Figures and Tables

**Fig. 1:**
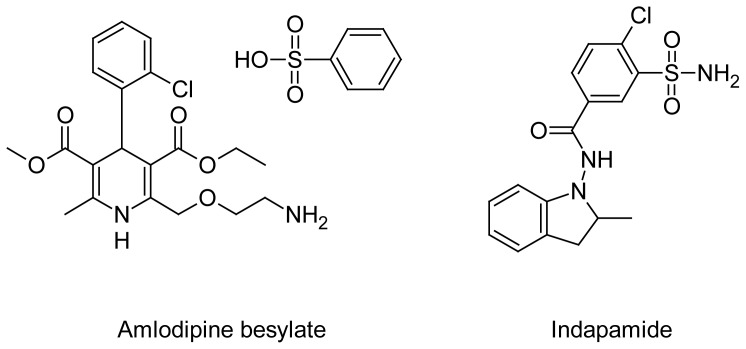
Structure of amlodipine besylate (AML) and indapamide (IND)

**Tab. 1:** System suitability test parameters for AML and IND at the proposed HPLC method

Parameters	AML	IND
Retention time (min)	5.6	3.9
Asymmetry	1.36	1.27
Resolution	2.0	
Theoretical Plates	4222	4322

**Tab. 2:** Regression analysis of calibration curves

Parameters	AML	IND
Range	0.25–35 (μg/ml)	0.075–10.5 (μg/ml)
Slope	37607	85909
SD of slope	101.25	739.78
Intercept	39026	33014
SD of intercept	33094.16	25772.7
Corr. Coefficient	0.9951	0.9945

**Tab. 3:** Data derived from accuracy study of the proposed method

Drug	Level	Amount taken (μg/ml)	Amount added (μg/ml)	Amount found (μg/ml)	% Recovery	RSD (n = 5)
AML	0%	5	–	5.08	101.6	1.65
	50 %	5	2.5	7.46	99.46	0.83
	100 %	5	5	9.96	99.6	1.96
	150 %	5	7.5	12.45	99.6	1.79

IND	0%	1.5	–	1.45	97.33	1.43
	50 %	1.5	0.75	2.29	100.88	1.02
	100 %	1.5	1.5	2.97	99	1.82
	150 %	1.5	2.25	3.72	99.2	0.64

**Tab. 4:** Robustness Studies

Method parameter/Condition	Deliberate changes	RSD of peak area (n=5)	

		AML	IND
Flow rate	0.8 ml/min	0.49	0.33
	1.2 ml/min	0.54	0.67

Mobile phase ratio 0.02 M KH_2_PO_4_:Methanol (total pH adjusted to 3 using *o*-phosphoric acid)	20:80(V/V)	0.48	0.75
	40:60(V/V)	0.84	1.10

**Tab. 5:** Summary of validation and system suitability parameters

Parameters	AML	IND
Retention time (min)	5.6	3.9
Asymmetry	1.36	1.27
Resolution	2.0	
Theoretical Plates	4222	4322
Detection limit (ng/ml)	2.9	0.99
Quantitation limit (ng/ml)	8.8	3
Accuracy(%)	99.46–101.60	99.46–01.60
Precision (%RSD)		
Intra-day (n=3)	1.23–1.78	1.06–1.86
Inter-day (n=3)	1.02–1.53	0.90–1.26
Instrument precision (%RSD)	0.18–0.63%	0.61–0.79%,
Robustness	robust	
Specificity	specific	

**Tab. 6:** Assay results of marketed formulation

Tablet Brand	Amount taken (μg/ml)		Amount found (μg/ml)		% Assay ± S.D. (n =5)	

	AML	IND	AML	IND	AML	IND
Amlodac-D	5	1.5	4.93	1.49	98.6 ± 1.55	99.3 ± 1.74
NAITRILAM	5	1.5	5.09	1.47	101.8 ± 1.65	98.0 ± 1.21
